# SWIFOLD: Smith-Waterman implementation on FPGA with OpenCL for long DNA sequences

**DOI:** 10.1186/s12918-018-0614-6

**Published:** 2018-11-20

**Authors:** Enzo Rucci, Carlos Garcia, Guillermo Botella, Armando De Giusti, Marcelo Naiouf, Manuel Prieto-Matias

**Affiliations:** 1 III-LIDI, CONICET, Facultad de Informática, Universidad Nacional de La Plata, La Plata (Buenos Aires), 1900 Argentina; 2 Depto. Arquitectura de Computadores y Automática, Universidad Complutense de Madrid, Madrid, 28040 Spain; 3 III-LIDI, Facultad de Informática, Universidad Nacional de La Plata, La Plata (Buenos Aires), 1900 Argentina

**Keywords:** DNA, Smith-Waterman, OpenCL, High-performance computing, FPGA

## Abstract

**Background:**

The Smith-Waterman (SW) algorithm is the best choice for searching similar regions between two DNA or protein sequences. However, it may become impracticable in some contexts due to its high computational demands. Consequently, the computer science community has focused on the use of modern parallel architectures such as Graphics Processing Units (GPUs), Xeon Phi accelerators and Field Programmable Gate Arrays (FGPAs) to speed up large-scale workloads.

**Results:**

This paper presents and evaluates *SWIFOLD*: a *S*mith-*W*aterman parallel *I*mplementation on *F*PGA with *O*penCL for *L*ong *D*NA sequences. First, we evaluate its performance and resource usage for different kernel configurations. Next, we carry out a performance comparison between our tool and other state-of-the-art implementations considering three different datasets. *SWIFOLD* offers the best average performance for small and medium test sets, achieving a performance that is independent of input size and sequence similarity. In addition, *SWIFOLD* provides competitive performance rates in comparison with GPU-based implementations on the latest GPU generation for the large dataset.

**Conclusions:**

The results suggest that *SWIFOLD* can be a serious contender for accelerating the SW alignment of DNA sequences of unrestricted size in an affordable way reaching on average 125 GCUPS and almost a peak of 270 GCUPS.

## Background

Biology, just like other scientific disciplines, is experiencing an exponential growth in data from experiments. Sequencing centers, analytical facilities and individual laboratories produce huge amounts of data, such as nucleotide and protein sequences, and this phenomenon is known as data explosion [1]. One of the main challenges for the scientific community is to extract relevant information from these data in a reasonable time, which has motivated the collaboration of disciplines such as Biology and Computer Science. One of the most useful operations in Bioinformatics is the identification of similarities between two biological sequences. To compute pairwise similarity, the Smith-Waterman (SW) algorithm is usually employed because of its high sensitivity. In fact, SW compares two sequences in an exact way and produces the optimal local alignment score. The complexity of SW depends on the input sequence lengths since the alignment process is of quadratic order. However, the main handicap of SW resides in the long execution times and computational resources required. This aspect has led to the use of BLAST [2] and FASTA [3], which, although they do not guarantee the optimal solution, are considerably faster. Despite the fact that heuristics are suitable in certain contexts, they do not always provide valid solutions due to a loss of sensitivity [4].

In order to reduce computational times, great efforts have been made to improve SW performance. Although a number of studies have focused on exploiting the different levels of parallelism that are now available on modern microprocessors, accelerating SW is still a big challenge. The parallelization of SW has been developed in two different alignment contexts: (i) a protein sequence against a genomic database; and (ii) two long DNA sequences. The first scenario involves the construction of a matrix of moderate size which allows the alignment of several independent sequences simultaneously [5]. However, in the context of DNA sequence, this scheme is impracticable due to limited memory resources. For example, in the DNA case, a single pairwise alignment of Megabase DNA sequences could involve a matrix size of petabyte scale. The parallelization approaches in DNA alignment are based on the *wavefront method* [6], in which the matrix is calculated by diagonals and all cells in each diagonal are computed in parallel.

In the last decade, we have seen countless parallel SW approaches in both contexts. Most of them correspond to protein alignment, and are parallelized on High-Performance Computing (HPC) architectures [7] and emerging architectures [8–10]. For very long sequences, such as with DNA, the number of works is significantly lower. CPU-based alternatives include the SSW library [11] and the recently released MASA framework [12] and Parasail library [13]. In the field of emerging architectures (especially hardware accelerators), two approaches that stand out are *SW#* [14] and *CUDAlign* [15], which compute the alignment of huge DNA sequences using multi CUDA-compatible GPUs. Also, there is an SW version for Intel Xeon Phi accelerators that is known as *mith-Waterman on Xeon Phi Clusters for Long DNA Sequences (SWAPHI-LS)* [16]. Furthermore, ad-hoc FGPAs proposals have shown significant speedups for DNA comparison [17–19].

However, in recent years we have observed significant transitions made by microprocessor manufacturers that will have a big impact on the HPC field. With the recent purchase of Altera by Intel in 2015, Intel has announced the incorporation of FPGA hardware in the next generation of Xeon processors. While there are studies in the transactional field that demonstrate great advantages in terms of performance and power consumption of large data centers equipped with these devices [20] (it is expected that more than 30% of data centers will be equipped with FPGAs), there is no study that confirms these advantages in other areas such as Bioinformatics. We would like to highlight that, unlike other accelerators such as GPUs or Xeon Phi, which have to be purchased separately, the new processors will integrate FPGA technology so that its exploitation will be essential to accelerate any research application. Traditionally, FPGAs were programmed at a low level using tools based on a hardware description language (HDL), which makes algorithm portability a very difficult and error-prone task. Recently, the main FPGA manufactures have introduced a high level programming paradigm known as Open Computing Language (OpenCL), which facilitates the portability process.

In the present study, we evaluate the performance of *SWIFOLD*, an SW implementation for DNA sequences of unrestricted size, on Intel’s FPGA by means of the OpenCL paradigm. Most existing studies into FPGA-based sequence alignment have been developed in HDL, and this limits their portability and design. We would like to point out that both Altera and Xilinx have promoted similar implementations in the past [21, 22], but no real sequence data were used and sequence lengths were fixed and very short, which can be radically different from real bioinformatic contexts. The focus of this paper is not only on the performance of Smith-Waterman Implementation on FPGA with OpenCL for Long DNA Sequences (SWIFOLD) but also on a guide to selecting the best existing option for a non-expert user. This work is an extension of the previous one published in [23], and the main contributions made here are:

- The creation of a public git repository with the binary executable developed for this paper, denoted as *SWIFOLD*^1^.
- The development of *SWIFOLD* and its optimization on Intel’s Arria 10 FPGA. The choice of Arria 10 is motivated by Intel’s announcement of the incorporation of Arria 10 FPGAs into both the new Xeon processors and the Intel-Go platform for automotive production at the 2017 Consumer Elec- tronics Association event (CES’2017). We would like to emphasize that the optimized code on the Arria 10 reported accelerations of between 3 × and 4 × in comparison with previous work [23].
- Additional experiments with larger DNA sequences than those used in [23]. This aspect emphasizes the independence of the *SWIFOLD* performance with regards to the sequence sizes.
- A useful guide to selecting the best platform for DNA sequence alignment. The selection depends on the matrix size and the sequence similarity, as well as the cost of the corresponding platform.

## Methods

### Smith-Waterman algorithm

The SW algorithm is widely used to obtain the optimal local alignment between two sequences [24]. This method is based on a dynamic programming approach and is highly sensitive since it explores all possible alignments between the sequences.

Given two sequences *S*_1_ and *S*_2_ of length |*S*_1_|=*m* and |*S*_2_|=*n*, the recurrence relations for the SW algorithm with affine gap penalties [25] are defined as follows:

1 $$ H_{i,j}=max \{0, H_{i-1,\,j-1}+SM(S_{1}[\!i],S_{2}[j]), E_{i,j}, F_{i,j}\}  $$

2 $$ E_{i,j}=max \{H_{i,j-1} - (G_{o}+G_{e}), E_{i,j-1} - G_{e}\}  $$

3 $$ F_{i,j}=max \{H_{i-1,\,j} - (G_{o}+G_{e}), F_{i-1,j} - G_{e}\}  $$

To identify a common subsequence, the similarity score *H*_*i*,*j*_ is computed. This contains the score for aligning the prefixes *S*_1_[1..*i*] and *S*_2_[1..*j*]. *E*_*i*,*j*_ and *F*_*i*,*j*_ correspond to the scores of prefix *S*_1_[1..*i*] and *S*_2_[1..*j*] aligned to a gap, respectively. The *scoring matrix* is denoted as *SM* and refers to match/mismatch scores between nucleotides. *G*_*o*_ and *G*_*e*_ represent the gap open and gap extension penalties, respectively. First of all, *H*, *E* and *F* must be initialized with 0 when *i*=0 or *j*=0. Then, the recurrences should be calculated with 1≤*i*≤*m* and 1≤*j*≤*n*. The highest value in the *H* matrix corresponds to the optimal local alignment score between *S*_1_[1..*i*] and *S*_2_[1..*j*]. The optimal local alignment is finally obtained by following a traceback procedure whose starting point is the maximum value in *H*.

**Fig. 1:**
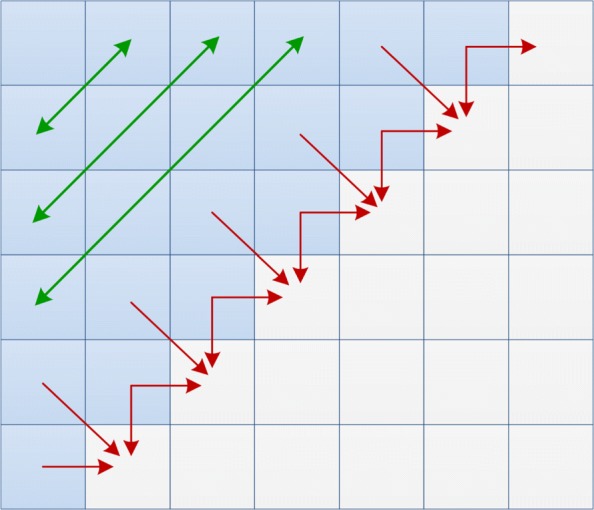
Data dependences in the alignment matrix *H*. Red arrows indicate the data dependences among cells while green arrows denote cells that can be computed simultaneously

From a computational point of view, it is important to highlight the computational dependences of any *H* element. Any cell can be calculated only after the values of the upper, left and upper-left neighbors are known (see Fig. 1). These dependences impose restrictions on the ways in which *H* can be processed.

### OpenCL extension on Intel’s FPGA

OpenCL is an extended framework for coding parallel programs across heterogeneous platforms. It offers a standard interface for parallel computing using task- and data-based parallelism. Currently, it is supported by most hardware devices such as CPUs, GPUs, Digital Signal Processors and FPGAs. Its definition and updated versions have been promoted by the Khronos Group consortium, in which most hardware vendors act as promoters.

OpenCL is based on the host-device model. While OpenCL routines called kernels are executed on the device, the host controls the device memory and the kernel code launch. Kernels can be seen as a piece of code which expresses the parallelism of a program. In this programming model, a program workload is divided into *work-groups* and *work-items*. While the task parallelism model exploits the parallelism between tasks following a pipeline scheme, the data parallelism model exploits the concurrent execution on different data (if non-dependence data exists). In the *task parallel* model, a kernel consists of a single *work-group* with a unique *work-item*. In the opposite sense, the *data parallel* model considers several *work-groups* composed of multiple *work-items*. These *work-groups* are executed independently on a processing element, usually in the Single Instruction Multiple Data (SIMD) manner.

The OpenCL memory model implements a particular memory hierarchy. Each region is distinguished by access type, scope and performance. Global memory is a high latency read-write memory accessible by all *work-items* and also by the host. Local memory is shared by all *work-items* in the same *work-group*. It can be seen as a scratchpad memory with low latency access. Private memory is only accessible by a single *work-item*. Constant memory, as its name suggests, is a read-only memory accessible by all *work-items*. In this sense, FPGAs are dedicated accelerators that obey the aforementioned complex hierarchy model (see Table 1 particularized for the FPGA used in this research).

**Table 1:** OpenCL memory model for the Intel Arria 10 FPGA and the resources available in the Arria 10 FPGA

	OpenCL	FPGA	Intel Arria 10 FPGA
*Memory*	Global	External	2GB DDR3
	Constant	Cache	32KB DDR3
	Local	Embedded	67Mbits
	Private	Registers	67244Kbits

One of the main advantages of OpenCL for a programmer is the abstraction of the target platform details in the parallel coding task. In fact, it favors portability and reduction of parallel coding effort. Note that FPGAs allow programming networks composed of logic elements, memory blocks and specific DSP blocks. HDLs are generally used to verify and create digital designs; however, they are complex and error prone, and have the additional difficulty of maintaining an explicit notion of time.

Each Intel FPGA can have multiple in-order command queues associated with it that can execute independent commands concurrently. Kernels need to be compiled previously using the Intel/Altera OpenCL Compiler (AOC). At the moment of selecting a parallel programming model, Intel FPGA OpenCL SDK [26] recommends the task parallel model as the best performance choice. We should point out that the AOC extracts efficient loop parallelism, which allows the loop to execute in a true pipeline fashion.

### SW implementation

The programming aspects and optimizations applied to our implementations on FPGA accelerated platforms are described in this section. For the sake of clarity, the pseudo-code for the host implementation is shown on Algorithm 1. Memory allocation and initialization are performed in OpenCL through *clCreateBuffer*, while memory transfer to the host is performed by means of *clEnqueueReadBuffer*. Finally, the *clEnqueueTask* function makes it possible to invoke kernel execution.


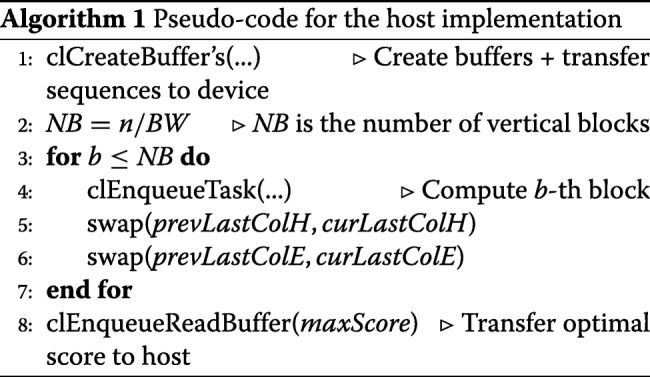



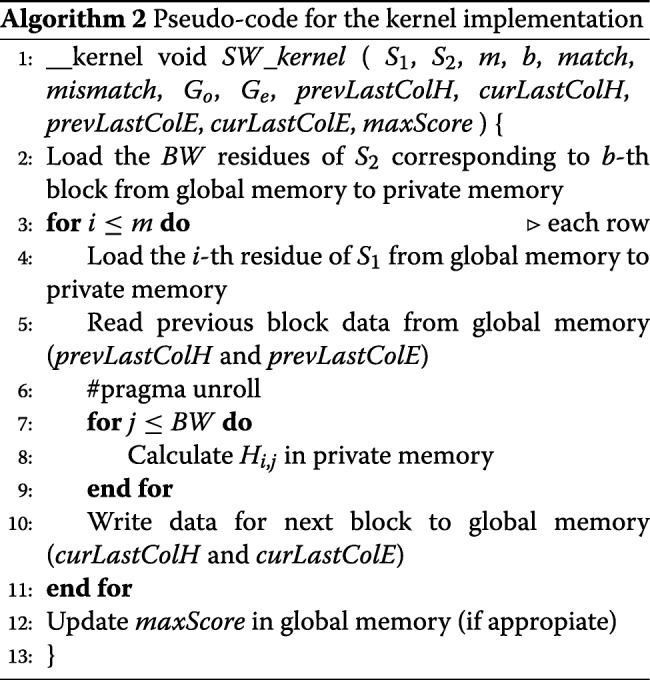


**Fig. 2:**
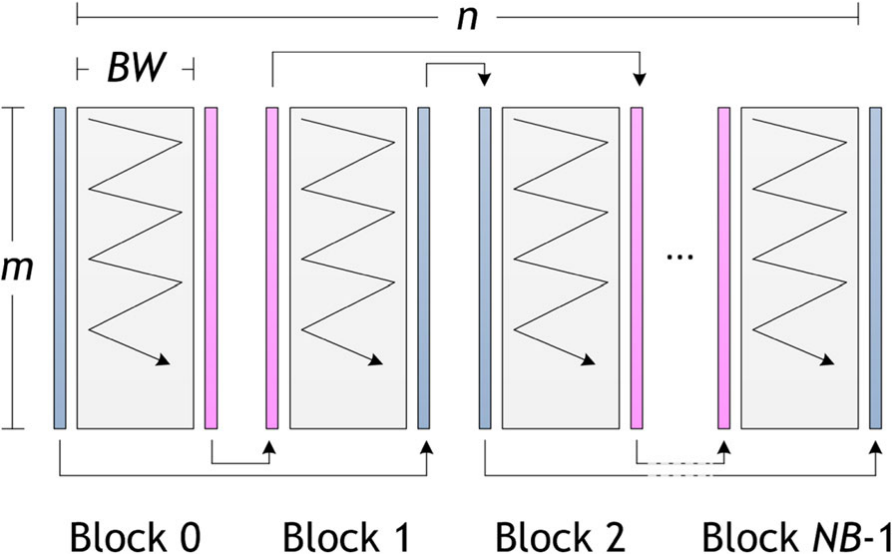
Graphic representation of our OpenCL kernel implementation

The task parallel programming model mentioned in “OpenCL extension on Intel’s FPGA” section is followed to implement the kernel, and its pseudo-code is presented in Algorithm 2. To reduce memory space requirements, the *H* matrix is divided into vertical blocks of size *BW*×*m* (*BW* means *B*lock *W*idth). Then each block is processed in row-by-row manner: from top to bottom, in a left to right direction, as is shown in Fig. 2. As well as improving data locality, this technique also favors the exploitation of the private low-latency memory. In this sense, we have used two buffers to store one row for matrices *H* and *F*. In addition, both sequences are partially copied to private memory.

From the performance point of view, fully unrolling the inner loop represents an essential aspect of this kernel since this technique allows the AOC to exploit loop instruction pipelining. As a consequence, the performance improves because more operations per clock cycle are carried out. As the compiler needs to know the number of iterations in the compile phase, the *S*_2_ sequence must be extended with dummy symbols to make its length a multiple of the fixed *BW* value. However, this extension has a negligible influence on execution time since DNA sequences are usually much larger than the *BW* constant. Furthermore, it is important to remark that the AOC reports the appearance of non-real read-write dependences in private memory associated to matrices *H* and *F* after a certain *BW* value, which aborts binary kernel generation. In order to solve this issue, the innermost loop is split into two or more loops to carry out the execution of wider blocks.

Global memory buffers are employed to solve the data dependences between adjacent vertical blocks mentioned in “Smith-Waterman algorithm” section, as each block needs the last column *H* and *E* values of the previous block. We used separate buffers to avoid read-write dependences in global memory: one for reading the values from the previous block and one for writing the values for the next block. After each kernel invocation, buffers are swapped in the host (these buffers are colored pink and blue in Fig. 2). It is important to mention that, although in the *OSWALD* implementation [10] Intel/Altera OpenCL channels are used to communicate these data, the use of this technique is not feasible in the context of DNA with millions of nucleotide bases involved, since its size would exceed by far the channel resources available. We should point out that although the use of these buffers could double memory consumption, it is by far compensated on speedup terms.

In addition, to improve data transfer efficiency, host-side buffers are allocated to be 64-byte aligned because the direct memory access mechanism is activated. Both sequences are copied when creating the device buffers and the optimal score is retrieved after all kernels are finished.

## Results and discussion

In this section, we describe the tests carried out and evaluate the performance of *SWIFOLD*. Additionally, we compare *SWIFOLD* with other existing alternatives and provide a guide to selecting the best platform for DNA sequence alignment according to the results obtained.

### Experimental platforms and tests carried out

The experiments were performed on three systems equipped with different accelerator types, namely FPGA, GPU and Xeon Phi. The main features of these systems are described in Table 2. All the tests were carried out with real DNA sequences from the National Center for Biotechnology Information (NCBI)^2^ in order to ensure the relevance of this study. The test sequences are divided into three sets: *small sequences* (less than 1M nucleotide bases, which generates kilo and mega-cell matrices), *medium sequences* (from 1M to 25M nucleotide bases, which generates giga-cell matrices), and *large sequences* (more than 25M nucleotide bases, which generates up to tera-cell matrices). The accession numbers and sizes of the sequences are presented in Table 3. For the sake of validation, optimal alignment scores were also included. The score parameters used were: +1 for match; -3 for mismatch; -5 for gap open; and -2 for gap extension. Finally, each test was run ten times and performance was calculated as an average of the corresponding execution times to avoid variability.

**Table 2:** Experimental platforms used in the tests

	Platform		
	FPGA	GPU	Xeon Phi
*Host*	2 ×Intel Xeon E5-2670 2.60Ghz	2 ×Intel Xeon E5-2695 v3 2.30Ghz	2 ×Intel Xeon E5-2695 v3 2.30Ghz
	(16 cores, 32GB RAM)	(28 cores, 64 GB RAM)	(28 cores, 128 GB RAM)
*Accelerator*	Intel Arria 10 GX	NVIDIA GTX 980	Intel Xeon Phi 3120P
		(Maxwell architecture, 2048 CUDA cores, 4GB RAM)	
	(2GB RAM)	NVIDIA GTX1080	(Knights corner generation, 57 cores, 6GB RAM)
		(Pascal architecture, 2560 CUDA cores, 8GB RAM)	
*Operating system*	CentOS release 6.5	Debian release 8.0	CentOS release 6.5
*Compiler*	Intel ICC 17.0.1.132	Intel ICC 17.0.1.132	Intel ICC 17.0.1.132
	Intel FPGA OpenCL SDK 16.0	CUDA SDK 7.5

**Table 3:** Information of the sequences used in the tests

Set	Sequence 1		Sequence 2		Matrix size	Score
	Accesion	Size	Accesion	Size	(cells)	
*Small*	AF133821.1	10K	AY352275.1	10K	100K	5027
	NC_001715.1	57K	AF494279.1	57K	3M	51
	NC_000898	162K	NC_007605	172K	28M	18
	NC_003064.2	543K	NC_000914.1	536K	291M	48
*Medium*	CP000051.1	1M	AE002160.2	1M	1G	82091
	BA000035.2	3M	BX927147.1	3M	9G	3888
	AE016879.1	5M	AE017225.1	5M	25G	5220775
	NC_005027.1	7M	NC_003997.3	5M	35G	157
	NC_017186.1	10M	NC_014318.1	10M	100G	10235056
	NT_033779.4	23M	NT_037436.3	25M	575G	9059
*Large*	NC_000021.9	48M	NC_006488.4	34M	1.6T	24922392
	NC_000022.11	51M	NC_006489.4	38M	1.9T	20133752
	NC_000019.10	59M	NC_006486.4	62M	3.7T	23570332
	NC_000020.11	65M	NC_006487.4	67M	4.4T	35488641

### Performance and resource usage evaluation

*Cell updates per second (CUPS)* is a commonly used performance measure in the SW scenario, because it makes it possible to remove dependency on the sequences utilized for the different tests. CUPS represents the time for a complete computation of one cell in matrix *H*, including all memory operations and the corresponding computation of the values in the *E* and *F* arrays. The billions of CUPS (GCUPS) is calculated with the formula $\frac {m \times n}{t \times 10^{9}}$, where *m* and *n* are the sizes of the sequences and *t* is the computation time. In this article, the runtime *t* includes device buffer creation, the transfer time of host data to the FPGA, the calculation time of SW alignment, and the transfer-back time of the optimal score.

We have considered different kernel implementations according to integer data type and *BW* value (see Table 4) to evaluate FPGA performance rates. The following items indicate the main differences:

- The name prefix denotes the integer data type used; i.e. *int*, *short* and *char* represent 32, 16 and 8 bit integer data types, respectively.

**Table 4:** Performance and resource usage comparison for the different OpenCL kernel implementations

Kernel		*int_bw256*	*int_bw512*	*int_bw1024*	*int_bw1152*	*short_bw512*	*short_bw1024*	*short_bw1536*	*char_bw512*	*char_bw1024*	*char_bw1536*
Integer type		int (32 bits)				short (16 bits)			char (8 bits)		
Maximum value		2147483647				32767			127		
BW		256	512	1024	1152	512	1024	1536	512	1024	1536
Resource	ALMs	29%	49%	87%	94%	32%	52%	73%	21%	31%	41%
usage	Regs	3%	3%	4%	4%	3%	4%	5%	3%	4%	4%
	RAM	8%	8%	20%	22%	7%	18%	27%	7%	18%	23%
	DSPs	0%	0%	0%	0%	0%	0%	0%	0%	0%	0%
Matrix size (cells)		Performance (GCUPS)									
	100K	24.15	31.57	44.99	49.81	48.00	52.35	56.92	-	-	-
	3M	34.94	61.59	101.89	105.14	80.71	122.72	160.44	93.03	152.75	223.1
	28M	36.70	68.11	119.15	122.91	85.96	146.80	186.74	102.50	173.23	255.49
	291M	37.32	69.23	122.32	126.95	87.18	149.90	195.17	105.14	181.16	268.83
	1G	37.42	70.13	124.93	129.44	-	-	-	-	-	-
	9G	37.84	70.80	126.96	131.45	88.40	155.85	202.56	-	-	-
	25G	37.91	70.92	127.49	131.96	-	-	-	-	-	-
	35G	37.93	70.94	127.47	131.98	88.71	156.43	203.51	-	-	-
	100G	37.98	70.99	127.68	132.15	-	-	-	-	-	-
	575G	38.03	71.09	127.85	132.33	88.87	156.83	204.06	-	-	-
- The name suffix denotes the *BW* value used; e.g. *bw256* means that the *BW* value was set to 256.

FPGA resource utilization and the performance achieved for our OpenCL kernel implementations using the small and medium test sets are shown in Table 4. *BW* impacts on both resource consumption and performance rates. As might be expected, larger *BW* values produce better performance results but at the expense of higher resource consumption. In fact, adaptive logic modules (ALMs) are the most affected resources: registers (Regs) and RAM blocks (RAMs) increase slightly, while DSP blocks (DSPs) remain intact. It is important to mention that, unlike in our previous work [23], we have been able to solve the non-real read-write dependences reported by the AOC for large *BW* values. This improvement allowed us to stress the kernel resources in order to maximize performance.

If we consider the integer data type, we can see that the use of a smaller data type generates better performance and less resource consumption. We can clearly appreciate this behavior when comparing the *int_bw512* and *short_bw512* kernels: for the same *BW* configuration, *short_bw512* presents an increment of up to 1.52 × in performance with a reduction of up to 0 − 0.35× in resource usage with regards to the *int_bw512* version. A similar behavior is observed with the *short_bw512* and *char_bw512* kernels: *char_bw512* reports an increment of up to 1.21 × in performance with a reduction of up to 0 − 0.35× in resource usage with regards to its *short_bw512* counterpart. Nevertheless, the use of narrower integer data types does not come free and involves an significant reduction in representation range. In this sense, there are three alignment scores out of ten that cannot be computed when using 16 bit integer data. This fact is also observed for the experiments with the 8 bit data type, where only three experiments could be carried out 3.

When considering sequence length, we can observe that larger workloads improve performance in all kernels regardless of sequence similarity. The best performances obtained are 132.43, 203.5 and 268.83 GCUPS for the *int*, *short* and *char* kernels, respectively.

### Performance comparison of *SWIFOLD* with other SW implementations

This subsection addresses a comparison of *SWIFOLD* with other SW implementations: the Xeon Phi-based *SWAPHI-LS* program (v1.0.12) [16], and the GPU-based *SW#* [14] and *CUDAlign* (v3.9.1.1024) [27] programs^4^. It is important to mention that we have also tested several CPU-based alternatives: the MASA/OpenMP implementation [12], and the SSW [11] and Parasail [13] libraries. However, we discarded all of these due to their poor performance rates. In particular, the best performances achieved using 2 ×Intel Xeon E5-2670 processors were 0.5, 2.42 and 1.3 GCUPS for MASA/OpenMP, SSW and Parasail, respectively.

Table 5 presents the performance of the *SWIFOLD*, *SWAPHI-LS*, *SW#* and *CUDAlign* implementations using the small and medium sequence test sets. It is worth noting that the *SWIFOLD* performance rates belong to the best 32-bit kernel version but faster performances for smaller data types are also reported (in brackets) where applicable. *SWAPHI-LS* yields an average performance of 25.89 GCUPS and a peak of 34.38 GCUPS, being outperformed by *SWIFOLD* in all scenarios. In particular, the most impressive performance difference occurs for the small sequence set where *SWIFOLD* runs on average 35.5 × faster. For the rest of the tests, the performance gain decreases but still improves by 4 × on average.

**Table 5:** Performance comparison among SW implementations using the *small* and *medium* sets

Implementation	SWIFOLD	SWAPHI-LS	SW#	CUDAlign	SW#	CUDAlign
Accelerator	Intel Arria 10 GX	Intel Xeon Phi 3120P	NVIDIA GTX980		NVIDIA GTX1080	
Matrix size (cells)	Performance (GCUPS)					
100K	49.81 (56.92)	0.42	0.3	0.03	0.23	0.03
3M	105.14 (223.1)	7.69	7.62	1.08	7.55	1.08
28M	122.91 (255.49)	21.24	33.33	8.18	41.47	8.63
291M	126.95 (268.83)	30.67	64.53	45.89	111.60	58.24
1G	129.44	32.84	75.24	79.21	144.97	117.97
9G	131.45 (202.56)	33.9	69.54	84.05	143.50	152.63
25G	131.96	34.16	120.92	160.79	255.89	295.43
35G	131.98 (203.51)	34.38	68.84	84.43	142.12	155.19
100G	132.15	33.19	118.81	163.77	253.13	297.05
575G	132.33 (204.06)	30.36	67.55	84.84	143.51	158.13

SWIFOLD performance rates belong to the best 32-bits kernel version but faster performances from smaller data types are also reported (between parenthesis) whenever correspond

Both GPU tools are very sensitive to sequence similarity since higher GCUPS are obtained on alignments with higher scores. On the GTX980, *SW#* presents an average performance of 62.68 GCUPS and a maximum performance of 120.92 GCUPS, improving upon *CUDAlign* by a factor of 5.34 × on average for the small dataset. *CUDAlign* achieves 71.23 GCUPS on average and a peak of 163.77 GCUPS on the medium test set, reaching an average speedup of 1.24 × with respect to *SW#* implementation. On the GTX1080, *SW#* obtains an average performance of 124.40 GCUPS, with a maximum performance of 255.89 GCUPS. On the same GPU, *CUDAlign* obtains 124.44 GCUPS on average, and 297.05 GCUPS as its peak. In a similar way, *CUDAlign* runs slower than *SW#* for the first half of the sequence alignments, for which the latter runs 4.3 × faster on average. For the second half of the test set, *CUDAlign* beats *SW#* by a factor of 1.12 ×. According to the results obtained for the GPU implementations, we can conclude that regardless of the GPU generation, for small sequences *SW#* performs better, whereas for medium sequences *CUDAlign* is slightly faster. For its part, *SWIFOLD* yields an average performance of 119.41 GCUPS, with a maximum performance of 132.33 GCUPS. In this way, *SWIFOLD* is able to beat *SW#* in all tests on the GTX980 and in the small test set on the GTX1080 (running 19.77 × and 58.77 × faster on average, respectively). Compared with *CUDAlign*, *SWIFOLD* is superior on the GTX980 by a factor of 201 × on average, except for the seventh and ninth alignments, for which *CUDAlign* performs better because of high sequence similarity. *SWIFOLD* outperforms *CUDAlign* when using the small dataset on both GPUs, and *CUDAlign* is superior for the medium dataset, but just on the most powerful GPU.

For larger dataset inputs, we have also compared the performance of the different tools. Table 6 presents the performance rates for the *SWIFOLD*, *SWAPHI-LS*, *SW#* and *CUDAlign* implementations. As might be expected, *SWAPHI-LS* again presents poor performance rates, obtaining 30.77 GCUPS on average and a peak of 33.66 GCUPS. *SWIFOLD* is able to beat both the *SW#* and *CUDAlign* implementations on the GTX980 GPU (1.5 × and 1.1 × faster on average, respectively), but this result changes for the most powerful GPU, on which the best performance is achieved by *CUDAlign*: 234.8 GCUPS on average (note the high score values reflected in Table 3).

**Table 6:** Performance comparison among SW implementations using the *large* set

Implementation	SWIFOLD	SWAPHI-LS	SW#	CUDAlign	SW#	CUDAlign
Accelerator	Intel Arria 10 GX	Intel Xeon Phi 3120P	NVIDIA GTX980		NVIDIA GTX1080	
Matrix size (cells)	Performance (GCUPS)					
1.6T	132.41	31.03	91.54	122.14	193.56	224.15
1.9T	132.41	27.86	84.93	110.77	180.34	231.91
3.7T	132.42	33.66	89.02	119.47	191.59	232.54
4.4T	132.43	30.41	95.61	132.22	138.22	250.78

### Best platform selection for DNA sequence alignment

From the results in the previous section, we can conclude that for the alignment of long sequences, such as in the case of DNA, the use of a general purpose processor is not the most suitable solution, considering the poor results achieved: MASA/OpenMP, SSW and Parasail libraries hardly obtain a maximum performance of 2.42 GCUPS. This fact forces us to use accelerators in order to obtain acceptable response times.

However, the choice of the optimal accelerator is not obvious since it involves an additional purchase. According to the results obtained, the use of a Xeon Phi accelerator does not seem to be an appropriate solution if we consider the performance on other accelerator types such as NVIDIA GPUs or Intel FPGAs.

The most successful implementations in this study are *CUDAlign* on the latest NVIDIA GPU and *SWIFOLD* on an Intel FPGA. The advantage of choosing a GPU lies in two aspects: the performance increment of successive GPU generations and their affordable prices. However, it is important to mention that newer GPU generations do not always provide better performance in the context of sequence alignments using the SW method, such as with CUDASW++ software [5]. Likewise, it has also been observed that *CUDAlign* does not always provide the best performance rates for small and medium sequence sizes. However, *CUDAlign* can be considered an efficient solution on the latest GPUs for large dataset inputs or very similar sequence pairs.

Taking into account the above analysis as well as Intel’s plans for FPGA integration into its next generation of processors, we consider that *SWIFOLD* can be a good choice for long DNA sequence alignment. *SWIFOLD* doest not only offer the best average performance for small and medium datasets, but also presents a performance that is independent of data input length and sequence similarity. Additionally, it is also competitive compared with *CUDAlign* on the latest generation of NVIDIA GPUs, running 1.6 × slower on average.

Finally, Table 7 summarizes the different SW implementations and the expected performance according to the alignment size and the accelerator type, where (+) and (-) mean better and worse options, respectively.

**Table 7:** Categorized options of SW implementations on different accelerator devices

Implementation	SSW	SWIFOLD	SWAPHI-LS	SW#	CUDAlign
Device	Intel multicore	Intel FPGA	Intel Xeon Phi	NVIDIA GPU	
Matrix size (cells)	Performance (GCUPS)				
Small	-	+++	+	++	+
Medium	-	+++	+	+++	+++
Large	-	++	+	++	+++

(+) and (-) mean better and worse options, respectively

## Conclusions

In this paper, we have presented and evaluated *SWIFOLD*. By using this tool, we have addressed the benefits of a parallel SW implementation using OpenCL on Intel FPGAs for DNA sequences of unrestricted size. By considering the performance of *SWIFOLD*, we can conclude that:

- Larger pipelines lead to better performance but at the cost of higher resource consumption. By splitting the innermost loop, we were able to avoid the non-real dependences reported by AOC and, as a consequence, stress the kernel resources in order to maximize performance.
- Data type exploitation has a significant effect on performance rates. Narrower data types reported better GCUPS with less resource usage, but at the expense of decreasing representation width.
- Larger workloads benefit all kernels regardless of sequence similarity. In particular, the fastest 32 bit kernel reached up to 132.43 GCUPS.
- Apart from the performance benefits, the use of the OpenCL paradigm for *SWIFOLD* programming facilitates the portability process, unlike the existing HDL-based alternatives.

If we compare *SWIFOLD* with other SW implementations on different devices and accelerators, we can conclude that:

- CPU-based implementations are not a suitable solution due to their unacceptable response times.
- With regards to Xeon Phi coprocessors, *SWAPHI-LS* reports the poorest performance rates.
- In the field of GPUs, *SW#* performs better for small sequences whereas *CUDAlign* is slightly faster for medium and large sequences, regardless of the GPU generation.
- For its part, *SWIFOLD* offers the best average GCUPS for small and medium test sets, its performance being independent of input size and sequence similarity. In addition, *SWIFOLD* reported competitive performance rates compared with *CUDAlign* on the latest GPU generation for the large dataset.

Furthermore, according to the results obtained and in view of the wide range of options, we have proposed a guide to selecting the best platform for DNA sequence alignment. As the choice is not obvious, the analysis provided can be helpful to a non-expert user at the moment of purchasing a computational platform. As a consequence of these promising results, the following aspects will be considered for future work:

- Since not all alignments require 32 bit integer data, and in order to look for the best performance-width trade-off, combinations of kernels with different integer data width will be considered.
- Since the chance of exploiting multiple devices is available in OpenCL, this work will be extended to a multi-FPGA environment in order to find the best workload distribution.
- As nowadays not only performance but power efficiency matters, we are interested in complementing the present study with a performance vs power analysis.

Finally, we would like to mention that the use of FPGAs for SW alignment has been traditionally limited by their programming cost and lack of portability. *SWIFOLD* solves these issues because it is a portable, parallel SW implementation for DNA sequences of unrestricted size on Intel FPGA-based platforms. As FPGAs are becoming increasingly popular and they are expected to be available on the next generation of servers, we expect *SWIFOLD* to become a serious contender for accelerating DNA alignment.

## Abbreviations

ALMs: Adaptive logic modules
AOC: Intel/Altera OpenCL compiler
CES’2017: Consumer electronics association event
CUPS: Cell updates per second
DSPs: DSP blocks
FGPAs: Field programmable gate arrays
GCUPS: Billions of CUPS
GPUs: Graphics processing units
HPC: High-performance computing
NCBI: National center for biotechnology information
OpenCL: Open computing language
RAMs: RAM blocks
Regs: Registers
SIMD: Single instruction multiple data
SW: Smith-Waterman
SWAPHI-LS: Smith-Waterman on Xeon Phi clusters for long DNA sequences
SWIFOLD: Smith-Waterman implementation on FPGA with OpenCL for long DNA sequences

## Footnotes

1 SWIFOLD is available at https://github.com/enzorucci/SWIFOLD.

2 Sequences are available at http://www.ncbi.nlm.nih.gov

3 The symbol ’-’ denotes an alignment that cannot be computed because the optimal score exceeds the corresponding maximum value.

4 Both GPU tools were configured to perform only their score version.
